# Effect of ω-3 and ω-9 fatty acid rich oils on lipoxygenases and cyclooxygenases enzymes and on the growth of a mammary adenocarcinoma model

**DOI:** 10.1186/1476-511X-9-112

**Published:** 2010-10-08

**Authors:** Andrea Comba, Damian M Maestri, María A Berra, Carolina Paola Garcia, Undurti N Das, Aldo R Eynard, María E Pasqualini

**Affiliations:** 1 Cátedra de Biología Celular, Histología y Embriología, Instituto de Biología Celular, Facultad de Ciencias Médicas, Universidad Nacional de Córdoba. Argentina; 2 Instituto Multidisciplinario de Biología Vegetal (IMBIV-CONICET), Cátedra de Química Orgánica, FCEF y N. Universidad Nacional de Córdoba, Argentina; 3 Consejo Nacional de Investigaciones Científicas y Técnicas (CONICET), Avenida Rivadavia 1917 - CP C1033AAJ - Qty. Buenos Aires, Argentina; 4 Jawaharlal Nehru Technological University, Kakinada 533 003, India; 5 UND Life Sciences, 13800 Fairhill Road, #321, Shaker Heights, OH 44120, USA; 6 Krishna Institute of Medical Sciences, Secunderabad-500 003, India

## Abstract

**Background:**

Nutritional factors play a major role in cancer initiation and development. Dietary polyunsaturated fatty acids (PUFAs) have the ability to induce modifications in the activity of lipoxygenase (LOX) and cyclooxygenase (COX) enzymes that affect tumour growth. We studied the effect of two diets enriched in 6% Walnut and Peanut oils that are rich in ω-3 and ω9 PUFAs respectively on a murine mammary gland adenocarcinoma as compared with the control (C) that received commercial diet.

**Results:**

Peanut oil enriched diet induced an increase in membrane arachidonic acid (AA) content and the cyclooxygenase enzyme derived 12-HHT (p < 0.05) and simultaneously showed decrease in 12-LOX, 15-LOX-2, 15-LOX-1 and PGE activities (p < 0.05) that corresponded to higher apoptosis and lower mitosis seen in this group (p < 0.05). Furthermore, Peanut oil group showed lower T-cell infiltration (p < 0.05), number of metastasis (p < 0.05) and tumour volume (p < 0.05) and longer survival rate compared to other groups.

**Conclusions:**

The results of the present study showed that Peanut oil-enriched diet protects against mammary cancer development by modulating tumour membrane fatty acids composition and LOX and COX enzyme activities.

## Introduction

Epidemiological studies showed that breast cancer incidence is increasing and is the third leading cause of death due to cancer [1]. Of all the environmental factors, nutrition has a significant role in the initiation and progression of breast cancer [2]. Dietary ω-3 and ω-6 polyunsaturated fatty acids (PUFAs) have been shown to play an important role in human breast, colon, prostate, pancreas, and stomach cancers [3]. Several studies suggested that ω-3 and ω-6 PUFAs are cytotoxic to different types of cancer cells and may act synergistically with current chemotherapeutic drugs [4].

Arachidonic acid (20:4, ω-6, AA) derived from the dietary essential fatty acid linoleic acid (18:2 ω-6, LA) can induce apoptosis of tumor cells by its ability to convert sphingomyelin to ceramide that triggers the release of pro-apoptotic proteins [5]. In addition, AA is converted by the catalytic activities of cyclooxygenase (COX), lipoxygenase (LOX) and cytochrome P450 (CYP450) enzymes to several eicosanoids that have potent biological actions [6]. Eicosanoids may act as active carcinogens or tumour promoters in view of their pro-inflammatory actions and by modulating the expression of various oncogenes and anti-oncogenes and thus, participate in cancer development [7,8]. Hence, inhibition or modulation of the AA cascade may suppress inflammatory events to bring about their anti-carcinogenic effects. Tumour cell-derived PGE_2 _inhibit the production of immune regulatory lymphokines, T-cell and B cell proliferation, and the cytotoxic activity of natural killer cells, thus favoring tumour growth [9]. Dietary manipulation of lipid sources may induce modification of PUFAs composition and physical properties of cell membranes that, in turn, influence eicosanoid synthesis and thus, affect tumour growth. Hence, we studied the effects of two dietary oils, one enriched in ω-3 and ω-6 from Walnuts and the other from Peanuts rich in ω-6 and ω-9 PUFAs on LOX and COX enzyme activities and their influence on the growth of a murine mammary gland adenocarcinoma and the results are reported here.

## Methods

### Plant material, oil extraction and analysis

Walnut (*Juglans regia *L. var. Chandler) and Peanut (*Arachis hypogaea *L., Runner market type) seeds were obtained from local markets of Argentina. Oils were extracted according to the procedure described by Tobares *et al. *[10]

### Tumour

Murine transplantable mammary adenocarcinoma (M3) in weaning BALB/c mice was used in the present study. M3 tumour is associated with 40% incidence of lung metastasis with a latency period of 6 ± 2 days after inoculation [11]. Three months after feeding the experimental animals with Walnut and Peanut oils, mice were inoculated with the tumour. Transplantation of tumours and other animal studies were conducted in accordance with the guidelines of the National Institutes of Health (NIH) Guide for the Care and Use of Laboratory Animals and all the procedures were approved by the Animal Research Committee of the Institute of Oncology A.H. Roffo, Buenos Aires, Argentina.

### Diets, Feeding Protocol, M3 tumour inoculum, Tumour Analysis

Three different isocaloric diets (caloric density 4,3 kcal/g) were used. The control group was fed on a commercial diet (GEPSA- Grupo Pilar, Argentina), considered as normal-fat diet (6% fat) for rodents containing ω-9: ω-6: ω-3 ratio = .1.4: 1.5: 0.1 The other two experimental groups were fed on a basic semi-synthetic diet, adjusted to the control diet in relation to nutrient content. The final composition of the experimental diets were: 6% tested oil, 17% casein, 33% sucrose, 38% corn starch, 2% fiber, 2% salt mixture and 0.5% vitamin mixture. The fatty acid (FA) oil composition is shown in Table 1. Diet 1 = supplemented with Walnut oil (Walnut) that has ω-9: ω-6: ω-3 ratio = 0.7: 1.7: 0.6; while diet 2 was supplemented with Peanut oil (Peanut) that has ω-9: ω-6: ω-3 ratio = 1.6: 1.4: 0.01.

**Table 1:** Fatty acids composition of Commercial diet, Walnut and Peanut oils. Fatty acids were determined as indicated in Methods section.

Diet	Saturated Fatty Acids						Unsaturated Fatty Acids						
							
							ω-7	ω-9			ω-6	ω-3	DBI/S
		
	14:0	16:0	18:0	20:0	22:0	24:0	16:1	18:1	20:1	22:1	18.2	18:3	
**Control**	0.65	18.41	5.78	0.0	0.0	0.0	3.00	33.27	0.0	0.0	36.54	2.34	4.68

**Walnut**	0.0	7.21	2.29	0.08	0.0	0.0	0.07	22.04	0.07	0.0	51.08	17.15	18.35

**Peanut**	0.0	9.34	1.20	0.65	2.23	1.18	0.0	43.56	2.62	0.46	38.45	0.30	8.92

Values are the average of at least three determinations (SEM was less than 6% in all cases). The degree of membrane FA unsaturation is expressed as DBI/S which means the sum of % each fatty acid × number of double bonds/% of saturated fatty acids.

Sixty post-weaned male and female BALB/c mice were randomly distributed among three groups (20 mice each). Food and water were provided *ad libitum*. Animals were kept in a light and temperature-controlled room. After three months of feeding with respective diets, mice were inoculated subcutaneously with 1 mm^3 ^of tumour tissue and were sacrificed 35 days after the inoculation of the tumor. At the end of the study, tumour volume was measured by a digital caliper and tumour cell (TC) suspensions were isolated from primary tumours for further studies [12]. The number of macroscopic metastasis was recorded in all the organs of the three groups of animals with the aid of a magnifying lens.

### Fatty acids analysis of oils and tumor cell membranes of animals fed Walnut and Peanut oils

Both Walnut and Peanut oils were subjected to alkaline saponification (1 N KOH) and the unsaponifiable matter was extracted with n-hexane and fatty acid methyl esters (FAME) were obtained using 1N sulfuric acid in methanol [13].

Tumor cell plasma membrane purification was achieved as described by Calderon et al [14]. Briefly, tumor cells (1 × 10^6^) were placed in homogenization solution (hypotonic Hepes-Manitol buffer) (Sigma-Aldrich, St. Louis, MO, USA) and homogenized using a Polytron (7 s. at setting 7). The homogenate was treated with 10 mM of CaCl_2 _and centrifuged at 3000 g for 15 min. The supernatant was saved and centrifuged at 48000 g for 30 min, and the pellet containing the plasma membrane fragments, was collected in deionized water and the lipids in the lower phase were extracted with chloroform: methanol: H_2_O (3:48:47 v/v). FAMEs were obtained with toluene and sodium metoxide at 4°C overnight [14].

The identification of the FAMEs was carried out by GLC using a capillary column of Polyethylene Glycol (30 m × 320 μm × 0.50 μm) (Phenomenex, Inc. U.S.A) using a Claurus 500 Perkin Elmers with an FID detector. FAME was identified by comparison of retention times with the corresponding commercial standards (Nu-Chek Prep, Inc. MN, U.S.A) [14].

### LOXs and COXs Enzyme Activities in Tumours

The activities of 12-LOX, 15-LOX-2 and COX-2 with AA as the substrate and 15-LOX-1 with LA as the substrate were estimated as described previously by Kelavkar UP et al, with slight modifications [15]. Briefly, tumour cell (TC) suspensions (1 × 10^7 ^TC/ml) were prepared from primary tumour that is free of necrotic areas, blood clots and connective tissue, with 0.01% pronase and 0.24% Type1-deoxyribonuclease in DMEM (Sigma-Aldrich, St. Louis, MO, USA) and were washed twice and resuspended in Ca^2+ ^and Mg^2+ ^free phosphate buffer saline (PBS) and stimulated with ionophore A23187 (2 M) (Sigma-Aldrich, St. Louis, MO, USA) for 15 min at 37°C. The metabolites were extracted using a STRATA C-18 cartridge (1 ml) (Phenomenex, Inc. U.S.A) and detected by Reverse-phase high performance liquid chromatography (HPLC). Analysis were conducted with a C18 Phenosphere-Next column (5 μm; 4.6 × 250 mm); (Phenomenex, Inc. U.S.A) equipped with a Beckman System Gold Programmable Module Model 126. Metabolic separation was achieved using a time program. A linear gradient from solvent A: methanol: water: acetic acid, 50:50:0.02 (v/v), pH 6 to solvent B: methanol, over 20 min. UV Programmable Detector Beckman System Gold Model 166 linked with a computer for data processing. UV analysis absorbance of the eicosanoid PGE_2 _was at 196 nm, and 12(S)-HHT, 12(S)-HETE, 15(S)-HETE and 13(S)-HODE was at 235 nm. Quantifications of eicosanoids PGE_2, _and 12(S)-HHT from COX-2 activity, and 12(S)-HETE, (12-LOX); 15(S)-HETE (15-LOX-2) and 13(S)-HODE (15-LOX-1) were obtained by using standard curves (Biomol International LP Plymouth Meeting, U.S.A) and expressed as (ng/1 × 10^7 ^TC) [16,17].

### Evaluation of Apoptosis and mitosis

Apoptosis of TC was analyzed by flow cytometry (FC) (Coulter^® ^XL EPICS^® ^Flow Cytometer), using an Annexin V- Fitc apoptosis detection kit (Sigma-Aldrich, St. Louis, MO, USA). The procedure is based on the binding properties of conjugated Annexin V-Fitc to plasma membrane phosphatidylserine (PS) in combination with propidium iodide (PI) staining [18]. A complementary approach to evaluate the same parameters was performed by counting the apoptotic and mitotic figures in slides of tumour tissue of 10 animals for each dietary condition *per *10 high-power fields scored descriptively or semiquantitatively in a blinded manner [12].

### Tumour leukocyte infiltration index

This study was assayed using CD3 complex expressed on mature T lymphocytes and NK-T cells in tumour tissue sections (4 μm) as the markers. The cells were washed with PBS and incubated with the CD3 monoclonal hamster antimouse antibody (1:100 PBS) (BD Biosciences Pharmingen, Philadelphia, U.S.A), Immunoreactivity was revealed using an avidin-biotin-peroxidase technique (Vectastain Elite ABC kit; Vector Laboratories, Burlingame U.S.A). Infiltrating leukocytes positive for CD3 were counted in high power fields at × 400 and expressed as cells *per *unit area ± SEM (*n *[microscopic fields *per *section] = 10 [19].

### Statistical analysis

Data obtained were analyzed using the ANOVA Test, and a generalized linear model with random component gamma, canonic link function (covariates) was used to evaluate the significance for diets, apoptosis, mitosis, eicosanoids, tumour leukocyte infiltration, tumour volume and metastases [20].

## Results

### Tumour cell membrane Fatty Acid Analyses

**Figure 1:**
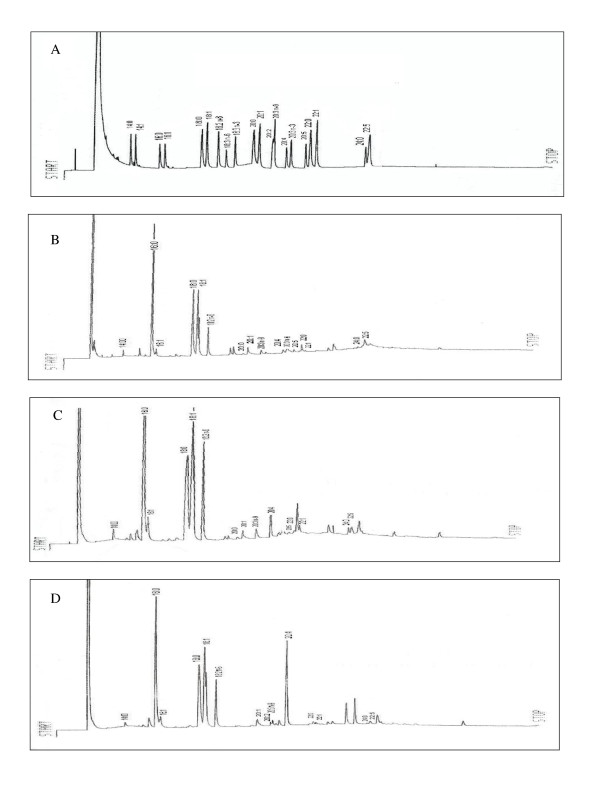
**Membrane Fatty acids profile of commercial standards A) and TC obtained from murine mammary adenocarcinoma cells of mice fed with Control diet B) or basic diet enrichment with Walnut oil C) or Peanut oil D)**.

The levels of AA found in TC membranes isolated from Peanut group was significantly higher (14.96%) than Walnut (3.15%) and Control (1.72%) (p < 0.05). Nevertheless, LA content found in Walnut TC membranes was higher (8.88%) than those measured in TC membranes from Peanut (8.22%) and Control (6.31%) groups (Fig 1 and Table 2).

**Table 2:** Fatty acid profile of TC membranes from host bearing M3 adenocarcinoma fed on different diets.

Diet	Saturated Fatty acids					Unsaturated Fatty Acid										
						
						ω-7	ω-9			ω-6			ω-3			DBI/S
		
	14:0	16:0	18:0	22:0	24:0	16:1	18:1	20:1	22:1	18:2	20:2	20:4	20:3	20:5	22:5	
**Control**	1.70	29:0	21.62	3.00	2.03	2.50	20.5	3.57	1.49	6.31	1.30	1.72	1.13	1.55	2.58	1.27

**Walnut**	1.50	27.35	21.66	1.83	2.80	1.65	21.45	1.92	1.16	8.88	1.75.	3.15	0.0	0.85	4.05	1.50

**Peanut**	0.92	23.70	18.14	1.66	0.77	2.79	22.60	2.09	0.71	8.33	2..21	14.96	0.0	0.00	1.13	2.50

Fatty acids composition of TC membrane was determined as indicate in Methods section. Values represent the mean in percentage of at least three determinations from TC suspensions of 10^8 ^cells/ml (SEM was less than 6% in all cases). The degree of membrane FA unsaturation expressed as DBI/S: sum of % each fatty acid × number of double bonds/% of saturated fatty acids.

The percentage ω-3 PUFAs in Peanut TC membranes was lower (1.13%) in comparison to Walnut (4.90%) and Control (5.26).

The degree of membrane FA unsaturation (DBI/S) of Peanut TC membranes was significantly higher (2.50) than Walnut (1.50) and Control (1.27) dietary treatment (Table 2).

### Effect of Diets on Tumour growth, Metastasis development and Animal Survival

**Figure 2:**
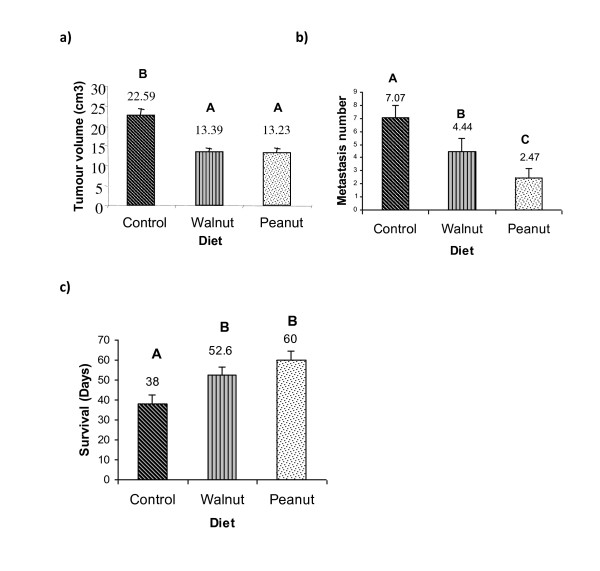
**Different parameters of tumour development and metastasis from M3 hosts fed on Control, Walnut and Peanut diets**. Different letters represent significant differences (p < 0.05): a) Tumour volume recorded during necropsy at 35 days after inoculation. Used as volume = tumour **height × width × height**. Values represent the mean ± SEM of 18 samples. b) Metastasis number of M3 host fed on diets. Values represent the mean ± SEM of 18 samples. c) Survival evaluation of M3 hosts fed on Control, Walnut and Peanut diets. Values represent the mean ± SEM of 10 animals. Different letters represent significant differences (p < 0.05).

Tumour volume from Peanut and Walnut group was lower (13.23 ± 1.24 cm^3 ^and 13.39 ± 1.08 cm^3^, respectively) than Control group (22.59 ± 1.6 cm^3^) (p < 0.05) (Figure 2-a). The number of metastases was lower in Peanut group (2.47 ± 0.67) than in Walnut (4.44 ± 1.05) and Control (7.07 ± 0.92) mice (p < 0.05) (Figure 2-b) and were mainly located in lung, peritoneum and liver.

Furthermore, mice from Peanut and Walnut groups showed the highest survival time (60 ± 4.92 and 52 ± 3.76 days after inoculum, respectively) compared to Control (38 ± 4.62 days) (p < 0.05) (Figure 2-c).

### Effects of Diets on tumour cell proliferation and apoptosis

**Figure 3:**
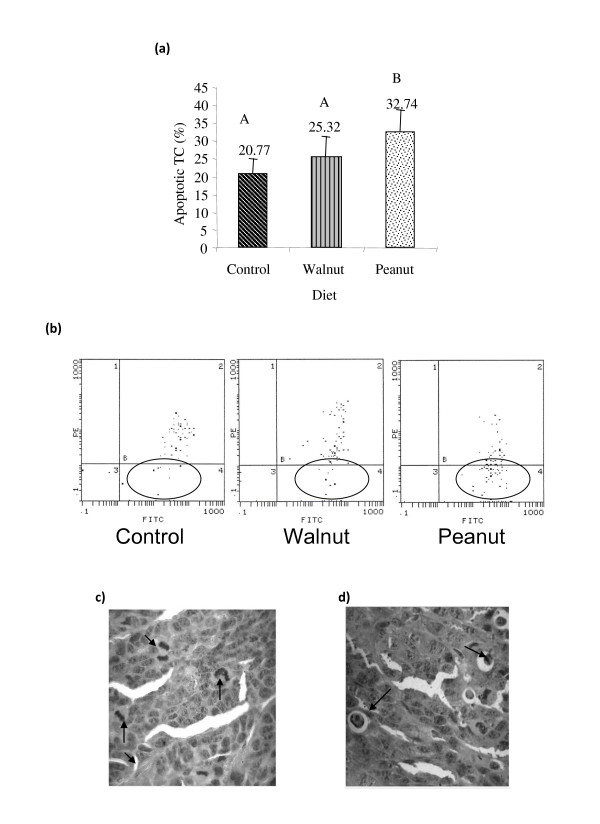
**a) Apoptotic cells in tumor cell suspension as determined by Flow cytometry using Annexin V/Propidium iodide double staining**. Values represent the mean ±SEM of six samples. Different letters represent significant differences (p**<**0.05).b) Flow cytometry graphics show the apoptotic cells populations in tumor cells suspensions in the different diet conditions (circle areas). c) Mitotic and d) apoptotic figures (arrows) on neoplastic tumor tissue fixed in 10% neutral formalin, dehydrated and embedded in paraffin and stained with hematoxylin and eosin (arrows, H&E, 400 ×).

As shown in Figure3 a and Figure 3b, TC from Peanut group showed significantly higher percentage of apoptotic cells (32.74 ± 5.99%) compared to Walnut (25.32 ± 6.12%) and Control groups (20.77 ± 4.27%) (p < 0.05). These results agree with the number of apoptotic figures recorded in tumour tissue: 2.03 ± 0.14/field; 1.40 ± 0.12/field and 1.16 ± 0.12/field in Peanut, Walnut and Control, respectively (p < 0.05) (Table 3). Moreover, the mitotic figures were inversely related to apoptotic values. We observed lowest mitosis values (1.56 ± 0.36/field) in Peanut group tumour tissue compared to Walnut and Control groups (2.62 ± 0.30 and 3.3 ± 0.25/field, respectively) (p < 0.05) (Table 3).

**Table 3:** Number of apoptosis and mitosis recorded in tumour sections from hosts fed on the different dietary conditions.

Diets	TUMOUR M3	
	
	Mitosis	Apoptosis
**Control**	3.3 ± 0.26**^A^**	1.16 ± 0.12**^A^**

**Walnut**	2.62 ± 0.30**^A^**	1.40 ± 0.12**^A^**

**Peanut**	1.56 ± 0.36**^B^**	2.03 ± 0.14**^B^**

Values represent the mean ± SEM of apoptotic and mitotic figures counted from 10 animals of each dietary condition (10 high-power fields). Different letters represent significant differences (p < 0.05).

### Modulation of LOX and COX Enzymes of the ω-6 Pathway in tumour cells

**Figure 4:**
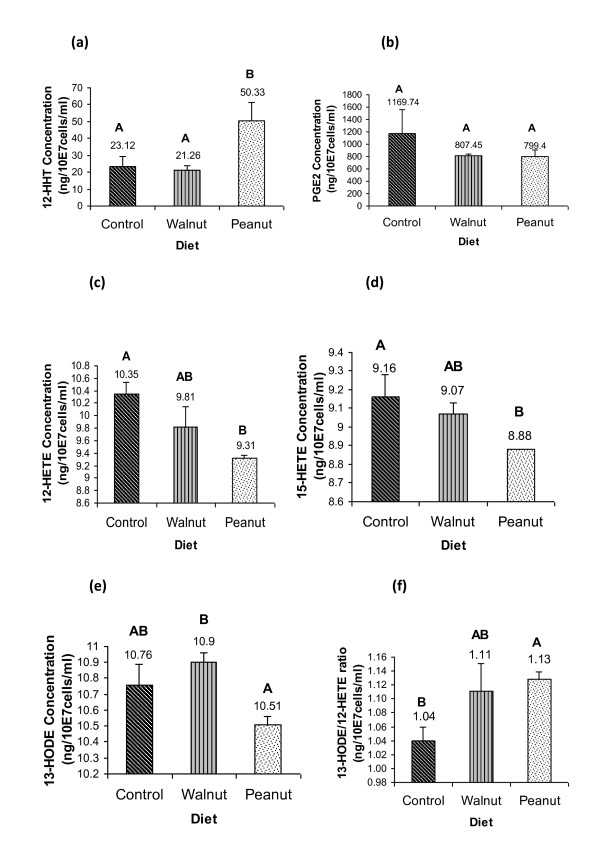
**Different eicosanoids released from M3 TC of hosts fed on different diets after stimulation with ionophore A 23287 (2 M)**. Values represent the means ± SEM of 15 samples. Different letters represent significant differences (p < 0.05): a) 12 (S)-HHT; b) PGE_2_; c) 12 (S)-HETE; d) 15 (S)-HETE; e) 13 (S)-HODE and f) 13 (S)-HODE/12 (S)-HETE ratio.

PUFAs of ω-6, ω-3 and ω-9 series modulated the formation and release of COX and LOX products of ω-6-AA and LOX products formed from w-6 LA after tumor cell stimulation with the ionophore A 23187 as shown in Figures 4. COX-derived-12(S)-HHT was significantly higher in Peanut group (50.33 ng) than Walnut (21.26 ng) and Control (23.12 ng) (Figure 4-a) (p < 0.05). On the other hand, PGE_2_, derived from the action of COX-2, released from the tumor cells of Control group were higher (1169.74 ng) compared with Peanut (799.40 ng) and Walnut (807.45 ng) groups (Figure 4-b), while tumor cells from the Peanut group released significantly lower levels LOX-derived AA eicosanoids: 12(S)-HETE (9.31 ng) and 15(S)-HETE (8.88 ng) compared with those released by tumor cells from Walnut (9.81 ng; 9.07 ng (p < 0.05) and Control groups (10.35 ng; 9.16 ng, respectively (p < 0.05) (Fig. 4-c and Figure 4d). 13 (S)-HODE product derived from LA by the action of LOX formed by the tumor cells of Peanut group was significantly lower (10.51 ng) compared to those formed from Walnut (10.90 ng) group. Though 13(S)-HODE formed by the tumor cells of the control group was higher compared to the Peanut group it was not significantly different (Figure 4e) Nevertheless, 13(S)-HODE/12(S)-HETE ratio was significantly higher in Peanut group (1.13) compared with both Walnut (1.11) and Control (1.04) groups (p < 0.05) (Figure 4-f).

### Effect of ω-6, ω-3 and ω-9 dietary PUFAs on Tumour leukocyte infiltration index

In order to know whether different oils could significantly influence tumor infiltrating leukocyte number, the number of lymphocyte T and NK-T cells in the tumor tissue was analyzed by CD3 inmunolabeling in tumour tissue sections (Table 4). The Peanut oil treated group showed the lowest leukocyte infiltration index (3.03 ± 1.05%) with respect to Walnut (16.71 ± 4.66%) and Control (29.23 ± 4.90%) (p < 0.05) (Table 4).

**Table 4:** Percentage of positive immunolabeling for CD3-T-lymphocytes and NK-T cells infiltration in tumor tissue.

Diets	% of Tumor Infiltration
**Control**	29.23 ± 4.90^A^

**Walnut**	16.71 ± 4.66^A^

**Peanut**	3.03 ± 1.05^B^

The values indicate immunolabeling and represent the mean ± SEM of gold-silver particles showing positive labeling of CD3 in 15 fields evaluated in 10 tumor sections of each dietary treatment. Different letters represent significant differences (p < 0.05).

## Discussion

Epigenetic factors have emerged as key mechanisms in cancer development. Of all the environmental factors, diet plays a critical role in the prevention and pathobiology of cancer [21]. In the present, we evaluated whether post-weaning diet of mice modulates LOXs and COXs activities through the eicosanoid release and their impact on certain parameters related to a mammary gland adenocarcinoma development. The comparative analysis of the three dietary conditions showed that mice fed on similar supplemented ω-6 fatty acids diets differing in ω-9 and ω-3 content showed that ω-9 enriched Peanut oil diet exhibited a protective effect on tumour development in comparison to the other dietary oil treatments. It was noted that Peanut oil group showed the lowest number of mitosis, the highest amount of apoptosis, decreased leukocyte infiltration and increased survival time (Table 3 and Table 4; Figure 2 and Figure 3).

Unexpectedly, as shown in Table 2, Peanut oil treated tumor cell membranes exhibited the highest ω-6 AA levels compared to Walnut and Control (25.50 *vs*. 13.78 and 9.33 respectively) groups despite the fact that (Table 1) these oils do not contain any AA, while LA content of Walnut oil is the highest of the three oils used in the study (see Table 1). It is also surprising to note that even though the Walnut treated tumor cell membranes contained significantly higher amounts of 22:5 ω-3 fatty acid, the tumor volume and metastasis number were higher, survival of animals bearing the tumors was lower, and tumor cell apoptosis was less and tumor cell mitotic numbers were higher in this group compared to the Peanut oil treated group. These results suggest that presence of higher amounts of ω-6 AA in the tumor cell membrane is responsible for the beneficial effects seen.

It is known that dietary ω-6 LA is converted to AA by the action of **Δ**6 and **Δ**5 desaturases and the corresponding elongases. It is likely that the high levels of AA noted in Peanut group may be linked to the up-regulation of **Δ6 **and **Δ**5 desaturases [21] that could explain the higher levels of unsaturated fatty acids in the tumor cell membranes [22] of Peanut oil treated group [23]. AA forms substrate to COXs and LOXs enzymes and some of their metabolites are: 12(S)-HHT and PGE_2 _*via *COXs; 12(S)-HETE and 15(S)-HETE *via *LOXs, while LA is a substrate for 15-LOX-1 and 13-HODE is its major metabolite. In the Peanut group, we observed the highest level of 12(S)-HHT, a lipoperoxide marker, and lower levels of PGE_2 _compared to control group. Both 12(S)-HHT and PGE_2 _are products of the activity of COX enzymes, whereas only PGE_2 _is formed due to the activity of the specific enzyme PGE synthase that explains changes in the levels of various eicosanoids in the cells. It has been shown that enhanced formation of lipid peroxides and low PGE_2 _production inhibit cancer progression and tumour growth [24]. Moreover, the lower percentage of tumour leukocyte infiltration exhibited in Peanut group (Table 4) correlated well with the low PGE_2 _levels observed, which is considered as an activator of inflammatory process and related to pro-carcinogenic events [25]. There is evidence to suggest that tumor infiltrating macrophages enhance tumor growth, promote tumor cell motility and angiogenesis [26,27]. Though the exact mechanism(s) by which tumor-infiltrating macrophages enhance tumor growth is not clear, it is possible that these macrophages produce excess of PGE_2_, TNF-α and other pro-inflammatory molecules that aid tumor cell growth. PGE_2 _is derived from AA, while TNF-α enhances PGE_2 _production [28]. On the other hand, PGE_2 _suppresses TNF-α production [29-32]. Inhibition of PGE_2 _synthesis was found to enhance TNF-α production and augment macrophage tumoricidal activity [29,31]. In contrast, phospholipase A_2 _activity and AA was reported to be essential for the tumoricidal action of TNF-α [33-36]. It is known that both ω-3 and ω-6 fatty acids inhibit the production of TNF-α [37-39]. Thus, the relationship among PUFAs, eicosanoids and TNF-α and their actions on tumor cells is complex. In this complex net work of events, in general, PGE_2 _serves as an immunosuppressor and inhibits the tumoricidal action of macrophages; TNF-α needs phospholipase A_2 _activity and free AA to bring about its anti-tumor action; while free AA and other PUFAs have direct tumoricidal action [28-41].

In the present study, we found that 12(S)-HETE levels were significantly lower, higher degree of apoptosis of tumor cells and lowest rate of mitosis counts in the (Figure 4 and Table 3) Peanut oil group suggesting that inhibition of 12(S)-HETE production diminishes cell proliferation and induces apoptosis as previously described [42]. Coincidently, high level of 12(S)-HHT could be related to increased apoptosis. These results are in agreement with previous studies that showed that lipid peroxides are selectively toxic to tumour cells by triggering apoptosis through nuclear caspase activation [43,44]. Indeed, oxidation is the initial mechanism for inducing the phosphatidyl-serine translocation from the cytosol domain to the external membrane, considered to be a key step in the apoptotic process [45]. Thus, lower tumor cell proliferation and enhanced apoptosis could have resulted in reduced tumour volume (Figure 2 and Figure 3). With respect to 15-LOX-1 activity, Peanut group showed significantly low levels of its major metabolite, 13-HODE. Also, this group exhibited the lowest metastasis number (Figure 2b). Although 13-HODE levels were lower in Peanut group, 13-HODE/12-HETE ratio was significantly higher (1.13) than Walnut (1.11) and Control (1.04) groups (Figure 4f). Previously, we showed that 13-HODE has anti-metastatic action, while 12(S)-HETE enhances metastasis [17]. In addition, it was observed that mammary gland tumour cells from mice fed with ω-6 enriched diet released higher levels of 12(S)-HETE which could be linked to the high number of metastasis in this animal model [12]. Several others showed that 12(S)-HETE is produced in large amounts by various epithelial cancer cell lines that positively correlated to their metastatic potential [46]. The lower amounts of 15(S)-HETE noted in Peanut oil fed tumor cell group could be attributed to its anti-tumour action lending support to the previous evidence that this metabolite is present in low concentrations in different tumour tissues [47].

In summary, the present study showed that diets enriched with high levels of ω-9 fatty acids reduce tumour growth, metastasis and tumor leukocyte infiltration by: 1) inhibiting LOXs activity, reducing the formation of pro-tumorigenic eicosanoids such as 12 (S)-HETE and 15 (S)-HETE, 2) increasing the synthesis of 12 (S)-HHT that induces apoptosis and 3) decreasing the production of pro-inflammatory PGE_2_. Understanding the mechanisms by which ω-3, ω-6 and ω-9 PUFAs alter growth and trigger apoptosis of breast cancer cells is essential to devise newer dietary therapeutic strategies to prevent cancer and employ various fatty acid rich oils to potentiate the actions of the current anti-cancer therapies.

## Competing interests

The authors declare that they have no competing interests.

## Authors' contributions

ARE and MEP conceived the study, participated in its design and coordination; AC, DMM, MAB and CPG performed various experiments; ARE, MEP and UND interpreted the data and wrote the manuscript; all authors read and approved the final manuscript
